# Structural and Functional Elucidation of IF-3 Protein of *Chloroflexus aurantiacus* Involved in Protein Biosynthesis: An *In Silico* Approach

**DOI:** 10.1155/2021/9050026

**Published:** 2021-07-01

**Authors:** Abu Saim Mohammad Saikat, Md. Ekhlas Uddin, Tasnim Ahmad, Shahriar Mahmud, Md. Abu Sayeed Imran, Sohel Ahmed, Salem A. Alyami, Mohammad Ali Moni

**Affiliations:** ^1^Department of Biochemistry and Molecular Biology, Bangabandhu Sheikh Mujibur Rahman Science and Technology University, Gopalganj 8100, Bangladesh; ^2^Department of Biochemistry and Molecular Biology, Gono University, Dhaka 1344, Bangladesh; ^3^Department of Biotechnology and Genetic Engineering, Islamic University, Jhenidah-Kushtia, Bangladesh; ^4^Department of Biochemistry and Molecular Biology, Jahangirnagar University, Dhaka 1342, Bangladesh; ^5^Department of Mathematics and Statistics, Faculty of Science, Imam Mohammad Ibn Saud Islamic University (IMSIU), Riyadh 13318, Saudi Arabia; ^6^School of Psychiatry, Faculty of Medicine, University of New South Wales, Sydney, NSW 2052, Australia; ^7^Healthy Ageing Theme, The Garvan Institute of Medical Research, Darlinghurst, NSW 2010, Australia

## Abstract

*Chloroflexus aurantiacus* is a thermophilic bacterium that produces a multitude of proteins within its genome. Bioinformatics strategies can facilitate comprehending this organism through functional and structural interpretation assessments. This study is aimed at allocating the structure and function through an *in silico* approach required for bacterial protein biosynthesis. This *in silico* viewpoint provides copious properties, including the physicochemical properties, subcellular location, three-dimensional structure, protein-protein interactions, and functional elucidation of the protein (WP_012256288.1). The STRING program is utilized for the explication of protein-protein interactions. The *in silico* investigation documented the protein's hydrophilic nature with predominantly alpha (*α*) helices in its secondary structure. The tertiary-structure model of the protein has been shown to exhibit reasonably high consistency based on various quality assessment methods. The functional interpretation suggested that the protein can act as a translation initiation factor, a protein required for translation and protein biosynthesis. Protein-protein interactions also demonstrated high credence that the protein interconnected with 30S ribosomal subunit involved in protein synthesis. This study bioinformatically examined that the protein (WP_012256288.1) is affiliated in protein biosynthesis as a translation initiation factor IF-3 of *C. aurantiacus*.

## 1. Introduction

In specific, *Chloroflexus aurantiacus* is a Gram-negative organism possessing exceptional characteristics, such as anoxygenic, filamentous, thermophilic, phototrophic, and gliding properties [1–3]. Keeping out other phototrophic anoxygenic, *Chloroflexus aurantiacus* sprout effectively in environments with a moderate temperature of 50–60°C [4, 5]. They can mostly acclimatize in various environmental circumstances, including wetlands, river water, hot springs, and sediments containing elevated-sulfide conditions [6, 7]. Surprisingly, the species of bacteria have specific similar characteristics, particularly chimeric photosystem, with purple-photosynthetic bacteria (PPB) and green-sulfur bacteria (GSB) [8, 9]. Placed in a certain way, the Chloroflexi are the first expanding bacteria that can generate their nutrients using photosynthesis [8]. Regarding the remarkable photosynthetic and thermophilic properties, the bacterium compelled investigators to examine multiple proteins involved with heat tolerance, formulating industrially crucial enzymes including propionyl-CoA synthase [10], maltotetraose-producing amylase [11], malonyl-CoA reductase, and so on in recent years [12]. Additionally, highlighting the genome's special features has attracted much attention by studying genome repositories.

Due to advances in computational biology, various platforms and methods have been built for predicting protein structure, recognizing sequence similarities performing phylogenic research, analyzing active site residue correlation, protein-ligand interaction, protein-protein interaction, gene expression screening, motif phosphorylation area recognition, and conserved domain determination [13–16]. A study using bioinformatics methods of the proteins allows one to evaluate their three-dimensional structural conformation, classify new domains, examine specific pathways to obtain a perspective of our evolutionary tree, identify additional clusters, and attach the proteins' role [17]. This accomplished knowledge can also impart effective pharmacological strategies and assistance in prospective drug design against many diseases [18–20].

The protein translation initiation factor IF-3 (WP_012256288.1) is deeply associated with protein biosynthesis in *C. aurantiacus*. The translation is the final phase of gene expression, which involves translating DNA into RNA and using the RNA to create amino acid chains. Translation includes four distinct stages. These phases include a pretranslational step, initial elongation, termination, and ribosome retrieval. Throughout each step, ribosomes interact with allied translation elements to relay signals essential for protein formulation. It is also crucial to know that the ribosome's conformational mechanisms, translation stimuli, and ribosomal complexes perform a crucial function in directing the translation system's directionality. A key obstacle for the scientists is to grasp how the poorly combined movements of the translational elements contribute to right and rapid protein synthesis [21]. IF-3 is one of the crucial elements required to stimulate the start of protein synthesis in prokaryotes. IF-3 attaches to the 30S ribosomal subunit (RS) and switches the balance between the 70S ribosomes and their available subunits in a manner that enhances the supply of free subunits, thereby maximizing the abundance of novel proteins ready to be constructed [22–24].

Additionally, this assessment enables the recognition of novel biotechnological targets through an adaptive mechanism that involves functional annotation, contemporary gene annotation, and three-dimensional protein modeling.

## 2. Methodology

### 2.1. Protein Selection and Sequence Retrieval

The amino acid (aa) sequence of the translation initiation factor IF-3 protein present in *Chloroflexus aurantiacus* was retrieved from the NCBI database (https://www.ncbi.nlm.nih.gov/) in FASTA format.

### 2.2. Physicochemical Characterization

The physicochemical parameters of the protein (WP_012256288.1) were evaluated by the ProtParam assessment tool of ExPASy server (https://web.expasy.org/protparam/) and the SMS v.2.0server (https://www.bioinformatics.org/sms2/).

### 2.3. Subcellular Location Identification

The subcellular location of the protein was documented by utilizing the CELLO v.2.5 [25, 26], PSORTb v.3.0.2 [27], SOSUI assessment tool [28], PSLpred server [29], HMMTOP v.2.0 [30], and TMHMM server v.2.0 (http://www.cbs.dtu.dk/services/TMHMM/).

### 2.4. Functional Annotation Prediction

The NCBI platform's CD search tool [31] was utilized to predict the conserved domain in the protein WP_012256288.1. Protein motif determination was performed using the GenomeNet (Motif) server [32], Pfam tool [33], and ScanProsite tool (https://prosite.expasy.org/scanprosite/) of the ExPASy program, and the SuperFamily program [34] assigned the evolutionary relationships of the protein WP_012256288.1.

### 2.5. Protein-Protein Interaction

The STRING v.11.0 program [35] was used for determining the possible protein-protein (pr-pr) interactions.

### 2.6. Secondary Structural Assessment

The SOPMA tool [36] utilized the secondary structural elements' prediction following the default parameters (window width of 17, number of states of 4, and the similarity threshold of 8) of the protein translation initiation factor IF-3 present *C. aurantiacus*. The SPIPRED v.4.0 [37] and the DISOPRED v.3.0 [38] tools were utilized to predict the secondary structure and the disordered areas, respectively.

### 2.7. Three-Dimensional Structure Prediction and Validation

HHpred predicted the three-dimensional (tertiary) structure with Modeller [39–41]. The most suitable template (HHpred ID: 5LMN_X; PDB ID: 5LMN) was selected for designing the tertiary structure among the number of hits of 130 with the probability, *E* value, aligned cols, and target lengths of 100, 2.5 × 10^−37^, 168, and 171, respectively. The PROCHECK [42] of the SAVES v.6.0 program (https://saves.mbi.ucla.edu/) was performed to predict the Ramachandran plot and validate the predicted tertiary structure.

### 2.8. Active Site Determination

The CASTp v.3.0 server [43] was used to predict the active sites of the modeled protein.

## 3. Results and Discussion

### 3.1. Sequence Retrieval

The amino acid (aa) sequence of the protein (WP_012256288.1) of *C. aurantiacus* was gathered from the NCBI database. The protein contains 275 amino acids. Further information on the protein (WP_012256288.1) is mentioned in Table 1.

### 3.2. Physicochemical Properties

Through studying the characteristics of each of the amino acids in the protein, it can be understood how the physicochemical features of the protein are defined. The ProtParam program of the ExPASy server was utilized to define the physicochemical properties of the protein (WP_012256288.1). The protein is consist of 275 amino acids where Arg (34) was the most abundant amino acid followed by Ala (33), Asp (33), Glu (29), Leu (20), Val (17), Lys (14), Gln (14), Pro (13), Ile (11), Thr (10), Gly (9), Ser (9), Asn (8), Phe (8), Met (7), Tyr (3), His (2), and Cys (1). There was no amino acid residue tryptophan (Trp) in the protein. Protein half-life is characterized as the period it requires for the radio-labeled focus protein density to be decreased by 50 percent compared to the amount at the onset of the chase [44]. The protein (WP_012256288.1) *C. aurantiacus* has an estimated half-life of about 30 hours (mammalian reticulocytes, in vitro), >20 hours (yeast, *in vivo*), and >10 hours (*Escherichia coli*, *in vivo*). The calculated isoelectric point (pI), molecular weight, and the total number of atoms were 4.88 (4.62^∗^), 31444.01 Dalton, and 4384, respectively (Table 2).

Besides, the molecular formula of the protein was C_1336_H_2179_N_417_O_444_S_8_. The pI value introduced the protein is negatively charged where the total number of negatively charged residues (Asp+Glu) was 62, and the total number of positively charged residues (Arg+Lys) was 48. Other parameters, including the instability index (II), describe the proteins' stability, whereas the aliphatic index (73.89) determines its balance over a broad temperature scale. The GRAVY index determines the proteins' solubility [45]. The negative value of GRAVY (-0.931) indicated the hydrophilic nature of the protein.

### 3.3. Subcellular Location Determination

The CELLO (v.2.5), PSORTb (v.3.0.2), SOSUI_Gram_N, and PSLpred tools were utilized for subcellular location assessment of the protein (WP_012256288.1). The tools predicted the subcellular location of the protein as a cytoplasmic protein. The HMMTOP (v.2.0) and TMHMM (v.2.0) programs predicted that there were no transmembrane helices in the protein (WP_012256288.1) and emphasized the cytoplasmic location of the protein present in *C. aurantiacus* (Table 3).

### 3.4. Functional Annotation of WP_012256288.1

The CDD tool of NCBI characterizes the domain that is found in the identical protein sequences. CD-Search employs RPS-BLAST to assess a test sequence across position-specific rating datasets that have been assembled from conserved domain (CD) alignments contained in the CD protein cluster.

The CD search tool predicted a conserved domain as a translation initiation factor IF-3 (infC, accession no. PRK00028) of the protein WP_012256288.1. IF-3 is one of the crucial elements for the onset of protein synthesis. It attaches to a 30S ribosomal subgroup, shifting the balance between 70S ribosomes and their 50S and 30S subgroups towards free subunits and thereby increasing the suitability of 30S subunits where protein synthesis activation starts. Besides, the ScanProsite program predicted a motif (position: 72–85; accession no. PS00938) as IF-3 (gene: infC), which is one of the primary elements required for protein biosynthesis in bacteria [46]. Also, the Pfam program described two different motifs at the positions of 98–181 (Pfam ID: IF3_C; IF-3, C-terminal domain; *e* value of 2.4 × 10^−34^) and 21–90 (Pfam ID: IF3_N; IF-3, N-terminal domain; *e* value of 4.0 × 10^−33^).

**Figure 1:**
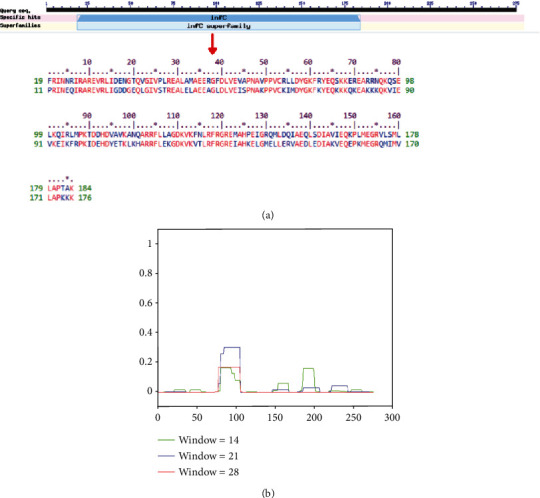
(a) Functional annotation of the protein WP_012256288.1. The graphical summary represents the conserved domains identified in the query sequence. The aligned sequences represent the conserved domains identified on the query sequence by comparing with the conserved protein domain family, infC (CDD accession no. PRK00028). The ScanProsite predicted a motif at 72–85 (accession no. PS00938) as infC, whereas the Pfam demonstrated two, including the C-terminal and N-terminal domain at 98–181 and 2–90 positions, respectively. The SuperFamily program predicted the protein as a member of the infC superfamily. Moreover, (b) coil reveals the heptads of existing windows 14 (green color), 21 (blue color), and 28 (red color). The *x*-axis of the diagram displays the adjustment in the protein of amino acid number (beginning at the N-terminus), whereas the *y*-axis indicates the spinning coil, while “window” corresponds to the width of the amino acid window, which is inspected concurrently.

The CDD tool also validated the domains IF3_C and IF3_N at 98–181 and 21–90. The IF3_C (CDD no. pfam00707) is the only member of the superfamily cl29551, whereas the IF3_N (CDD no. pfam05198) is the only member of the superfamily cl04980 as of the conserved protein domain family search feature by the CDD program. The SuperFamily tool predicted the protein WP_012256288.1 (Figure 1) as profoundly associated with the infC superfamily (*e* value of 2.09 × 10^−98^). The *x*-axis of the diagram displays the location in the amino acid (aa) count protein (beginning at the N-terminus), and the *y*-axis indicates the coiled coil, while the “window” corresponds to the amino acid window which is examined simultaneously (Figure 1).

### 3.5. Protein-Protein Interaction

The primary focus of protein-protein interactions is acknowledging how cellular systems operate. Such connections allow the filtering, evaluating, and validating of functional genomics data and offering an insightful platform for annotating functional, structural, and evolutionary features of proteins.

**Figure 2:**
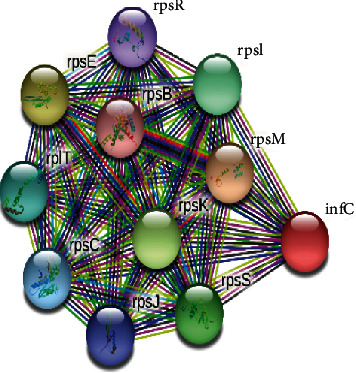
The STRING network of the protein determines the protein-protein interactions. The rpsM, rpsE, rpsK, rpsS, rpsI, rpIT, rpsC, rpsJ, rpsR, and rpsB represent the 30S ribosomal protein S13, 30S ribosomal protein S5, 30S ribosomal protein S11, 30S ribosomal protein S19, ribosomal protein S9, 50S ribosomal protein L20, 30S ribosomal protein S3, 30S ribosomal protein S10, 30S ribosomal protein S18, and ribosomal protein, respectively. Colored nodes represent query proteins and the first shell of interactors, and white nodes describe the second shell of interactors. For node content: empty nodes designate proteins of unknown 3D structure, and filled nodes render some 3D structure as known or predicted.

The platform can furnish predictions for prospective experiments and map the interactions between different species [47]. The STRING v.11.0 program was performed to determine the protein-protein (pr-pr) interaction. The STRING program determined the functional fellows with scores as of rpsM (0.990), rpsE (0.988), rpsK (0.988), rpsS (0.987), rpsI (0.983), rpIT (0.980), rpsC (0.980), rpsJ (0.964), rpsR (0.955), and rpsB (0.951). The rpsM, rpsE, rpsK, rpsS, rpsI, rpIT, rpsC, rpsJ, rpsR, and rpsB are the 30S ribosomal protein S13, 30S ribosomal protein S5, 30S ribosomal protein S11, 30S ribosomal protein S19, ribosomal protein S9 which belongs to the universal ribosomal protein uS9 family, 50S ribosomal protein L20, 30S ribosomal protein S3, 30S ribosomal protein S10, 30S ribosomal protein S18, and ribosomal protein S which belongs to the universal ribosomal protein uS2 family, respectively (Figure 2).

### 3.6. Secondary Structure Inquiry

**Figure 3:**
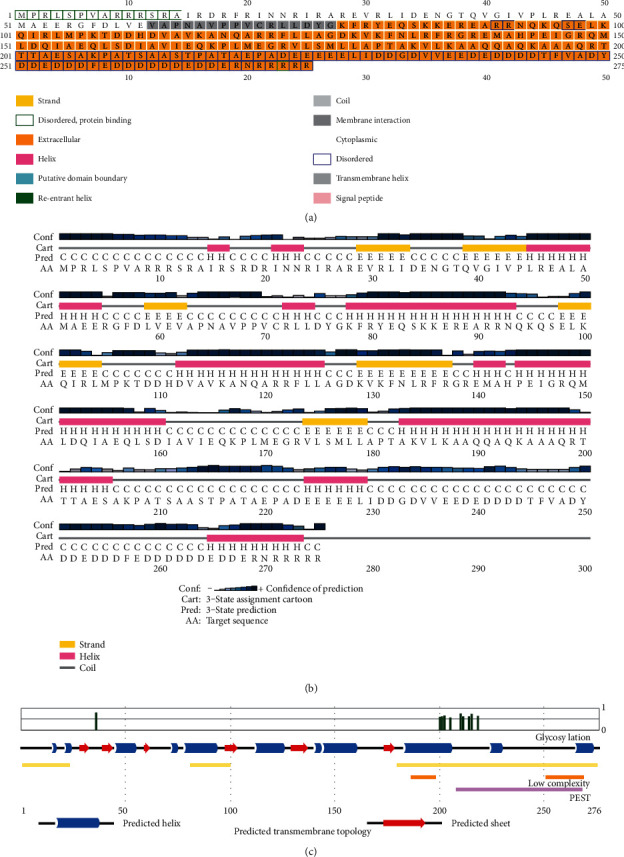
The secondary structural assessment: (a) sequence plot, (b) the predicted secondary structure, and (c) predicted transmembrane topology (position-dependent feature predictions are mapped onto the sequence schematic phenomena; the line height of the phosphorylation and glycosylation features reflects the confidence of the residue prediction).

Protein structure and function are strongly connected. The secondary structural components, e.g., helix, coil, sheet, and turn, have an excellent relationship with protein function, structure, and engagement [48, 49]. The SOPMA program predicted the secondary-structural element of the protein (WP_012256288.1) where the alpha helix (Hh), extended strand (Ee), beta turn (Tt), random coil (Cc) were 121 (44.00%), 45 (16.36%), 23 (8.36%), and 86 (31.27%), respectively (Table 4). The SPIPRED v.4.0 and DISOPRED v.3.0 tools predicted the sequence plot, secondary structure, and transmembrane topology (Figure 3). The sequence plot from the secondary structure of the IF-3 protein (Figure 3(a)) represents that most of the protein is extracellular, whereas Table 3 reports the protein as cytoplasmic. Further studies are required to unleash the nature of the protein.

### 3.7. Tertiary-Structure Prediction and Validation

**Figure 4:**
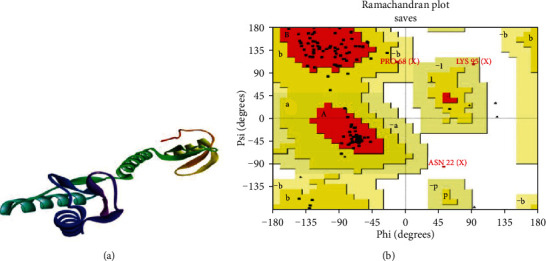
Tertiary structure prediction. (a) Predicted tertiary structure by HHpred tool employing the Modeller application. (b) The Ramachandran plot statistics of the modeled three-dimensional structure validated by the PROCHECK program.

Homology modeling (HM) is a primary method for estimating protein architecture when solely amino acid sequence information is accessible. Protein activities can be derived from the composition of the chain. Using homology modeling (HM) or comparative modeling (CM), scientists would quickly evaluate two closely related sequences' similarities and roles. Sequence similarity to a defined structure is typically representative of translational and structural similarities to that structure. In the face of these constraints, sequence similarity below 30% will never provide suitable efficiency in structure prediction [50, 51]. The HHpred is a powerful platform used for distant homology identification and structure estimation, implemented initially as hidden Markov models (HMMs), pioneered by the earliest pairwise comparative analysis of homologous protein profiles. It enables a broad range of repositories, including PDB, Pfam, SCOP, COG, SMART, and CDD. It admits a solitary query array or multiple lineups as input, and it delivers the findings to a PSI-BLAST-like user-friendly interface. Search features are including local or global integration and the detection of secondary systems. HHpred can generate a pair of query prototypes, multiple model alignments with several frameworks from the lookup findings, and 3D structural models from these configurations computed with the Modeller program [52]. The HHpred prognosticated the three-dimensional structure of WP_012256288.1 employing the Modeller application (Figure 4). The template (HHpred ID: 5LMN_X) [53] for modeling the three-dimensional structure was chosen based on the most similarity with the IF-3 protein sequence.

The SAVES server's PROCHECK program was utilized for structural quality assessment of the modeled protein, where the arrangement of the *ψ* angle and the *φ* angle is shown (Table 5, Figure 4). Residues in the most favored regions engulfed 92.0%, which validated the protein's modeled tertiary structure (WP_012256288.1). Also, residues in additional allowed regions generously allowed regions, disallowed regions, no. of nonglycine and nonproline residues, and no. of end residues (excl. Gly and Pro) were 10 (6.7%), 1 (0.7%), 1 (0.7%), 150, and 2, respectively. The no. of glycine residues and the no. of proline residues were similar (8 residues) found in the protein 3D structure. The C-terminal portion of the protein IF-3 appeared irregular as it contained high charge and repeated regions (Figure 4(a)). Further investigations for describing the functions are required to reveal the mystery, whether due to translation error and/or being a member of the same family.

### 3.8. Active Site Determination

**Figure 5:**
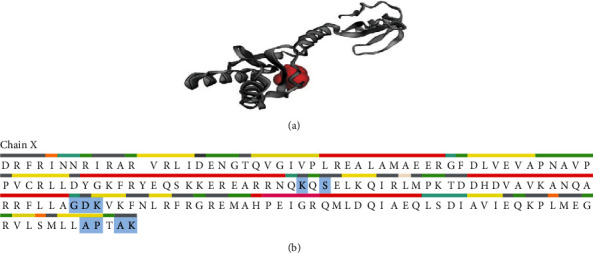
Active site determination: (a) the amino acid residues in the active site (blue color) and (b) active sites of the protein (WP_012256288.1). Also, the “red sphere” indicates the active sites of the protein.

The CASTp v.3.0 program predicted 21 different active sites of the modeled protein (Figure 4). CASTp is a database server that can locate areas on proteins, delineate their outline, find the areas' dimensions, and calculate the regions' area. This involves pockets on protein surfaces and vacuums concealed within proteins. The calculation consists of a pocket and volume spectrum or vacuum, both mathematically determined by a solvent-accessible surface (surface of Richards) and molecular surface model (surface of Connolly). CASTp could be utilized for the investigation of surface properties and protein operational zones. CASTp provides a pictorial, user-interface versatile, dynamic view and user-submitted constructs on-the-fly measurement [43]. The top active sites of the modeled protein were identified between the area of 85.302 and the volume of 50.667 (Figure 5).

## 4. Conclusions

Comprehending how proteins act is essential for explaining how they operate, and this protein contains IF-3, a crucial factor in protein synthesis considered to initiate protein synthesis. IF-3 connects to the 30S ribosomal subunit and alters the balance between the 70S ribosomes and their 50S and 30S subunits, thereby strengthening the abundance of the 30S subunit's affordability of amino acids for the initiation of protein biosynthesis. This investigation reveals the fundamental characteristics including cytoplasmic nature and functional annotation of the protein in association with tertiary structure. Thus, the study findings show the efficiency and scale of further studies on the IF-3 protein of bioinformatics methods used in this investigation.

## Figures and Tables

**Table 1:** Protein retrieval.

Protein individualities	Protein information
Locus	WP_012256288
Amino acid	275 aa
Definition	Translation initiation factor IF-3 [*Chloroflexus aurantiacus*]
Accession	WP_012256288
Version	WP_012256288.1
Source	*Chloroflexus aurantiacus*
Keywords	RefSeq
Organism	*Chloroflexus aurantiacus*
FASTA sequence	>WP_012256288.1 translation initiation factor IF-3 [*Chloroflexus aurantiacus*] MPRLSPVARRRSRAIRDRFRINNRIRAREVRLIDENGTQVGIVPLREALAMAEERGFDLVEVAPNAVPPVCRLLDYGKFRYEQSKKEREARRNQKQSELKQIRLMPKTDDHDVAVKANQARRFLLAGDKVKFNLRFRGREMAHPEIGRQMLDQIAEQLSDIAVIEQKPLMEGRVLSMLLAPTAKVLKAAQQAQKAAAQRTTTAESAKPATSAASTPATAEPADEEEEELIDDGDVVEEDEDDDDTFVADYDDEDDDFEDDDDDDEDDERNRRRRR

**Table 2:** Physicochemical parameters.

Parameters	Value
Molecular weight	31444.01
Theoretical pI	4.88, 4.62^∗^
Total number of negatively charged residues (Asp+Glu)	62
Total number of positively charged residues (Arg+Lys)	48
Formula	C_1336_H_2179_N_417_O_444_S_8_
Total number of atoms	4384
The estimated half-life	(a) 30 hours (mammalian reticulocytes, *in vitro*) (b) >20 hours (yeast, *in vivo*) (c) >10 hours (*Escherichia coli*, *in vivo*)
Instability index (II)	60.38
Aliphatic index	73.89
Grand average of hydropathicity (GRAVY)	-0.931

^∗^pI calculated by the SMS v.2.0.

**Table 3:** Subcellular localization assessment.

Analysis	Result
CELLO (v.2.5)	Cytoplasmic
PSORTb (v.3.0.2)	Cytoplasmic
SOSUI_Gram_N	Cytoplasmic
PSLpred	Cytoplasmic
HMMTOP (v.2.0)	No transmembrane helices present
TMHMM (v.2.0)	No transmembrane helices present

**Table 4:** Secondary structural elements.

Structural elements	Values (%)
Alpha helix (Hh)	121 (44.00)
3_10_ helix (Gg)	0
Pi helix (Ii)	0
Extended strand (Ee)	45 (16.36)
Beta bridge (Bb)	0 (0.00)
Bend region (Ss)	0 (0.00)
Beta turn (Tt)	23 (8.36)
Random coil (Cc)	86 (31.27)
Ambiguous states	0
Other states	0

**Table 5:** Ramachandran plot statistics of the modeled protein.

Ramachandran plot statistics	Value (%)
Residues in the most favored regions [*A*, *B*, *L*]	138 (92.0)
Residues in additional allowed regions [*a*, *b*, *l*, *p*]	10 (6.7)
Residues in generously allowed regions [~*a*, ~*b*, ~*l*, ~*p*]	1 (0.7)
Residues in disallowed regions	1 (0.7)
Number of nonglycine and nonproline residues	150
Number of end residues (excl. Gly and Pro)	2
Number of glycine residues (shown as triangles)	8
Number of proline residues	8
Total number of residues	168

## Data Availability

The data used to support the findings of this study are available from the submitting or corresponding author on request.

## Abbreviations

NCBI: National Center for Biotechnology Information
PDB: Protein Data Bank
SMS: Sequence Manipulation Suite
CDD: Conserved Domain Database
RPS-BLAST: Reverse Position-Specific BLAST
BLAST: Basic Local Alignment Search Tool
GRAVY: Grand average of hydropathicity
CELLO: Subcellular localization predictor
PSLpred: Prediction of subcellular localization of bacterial proteins
HMMTOP: Prediction of transmembrane helices and topology of proteins
SPIPRED: PSI-blast based secondary structure prediction
SOPMA: Self-optimized prediction method
CASTp: Computed atlas of surface topography of proteins.
